# Actualizing community–academic partnerships in research: a case study on rural perinatal peer support

**DOI:** 10.1186/s40900-022-00407-0

**Published:** 2022-12-18

**Authors:** April Hards, Audrey Cameron, Eva Sullivan, Jude Kornelsen

**Affiliations:** 1 Golden, Canada; 2 Centre for Rural Health Research, Department of Family Practice, University of British Columbia, 3rd Floor David Strangway Building, 5950 University Boulevard, Vancouver, BC V6T1Z3 Canada

**Keywords:** Collaborative research, Patient partner involvement, Co-production, Perinatal, Mental health, Rural, Maternity, Community partner, Co-design

## Abstract

**Background:**

Within the field of patient and public involvement in health service research, there is a growing movement towards not only involving patients in research but engaging them as *co-producers* of knowledge. We explore such a co-productive research relationship in a case study on rural perinatal mental health, with the aim of collaboratively developing knowledge based on both the relevant lived experience of a community partner, and the systemic knowledge of academic researchers.

**Methods:**

Data was gathered through a community forum and subsequent interviews with social service program administrators from rural British Columbia, Canada. Interviews were analyzed separately by the community partner and academic researchers using principles of thematic analysis. Both the community partner and academic researchers were involved from project genesis to data collection, analysis, interpretation, and manuscript writing.

**Results:**

Common themes identified by the academic and community researchers included needs for peer support, barriers to peer support, and gaps in mental health care. Divergently, the academic researcher focused on systems-level challenges while the community partner emphasized the impact of power dynamics within health systems. Researchers generated five methodological values propositions from the process of co-production, including (a) mutual respect for all viewpoints, (b) a rejection of assumed hierarchy, (c) commitments to truth speaking, (d) attention to process, and (e) equivalence of contribution.

**Conclusions:**

Co-production highlights the value of lived experience in health research, sets it in conversation with scientific inquiry, and moves away from hierarchies of assumed knowledge often embedded in traditional health care research. Incorporating both academic researcher and community partner writing into our paper reflects a commitment to maintaining the integrity and authenticity of lived experience, an affirmation of its equal validity as a source of knowledge, and a rejection of qualifying patient voices. The exploration of this co-production research relationship lays groundwork for future research teams considering collaborative methodology. We suggest co-productive research as a means of addressing the epistemic injustice that arises in health care research from the privileging of certain forms of knowledge, and the exclusion of others, namely that derived from patient experience.

## Plain English summary

Co-production is an approach to research where community partners and academic researchers work together to carry out a study. Our co-production team was made up of a community partner with lived experience of accessing mental health supports in rural areas, and academic researchers experienced in health systems design. Co-production emphasizes both the wisdom of lived experience and the importance of scientific approaches. What emerges is research that is both rigorous and authentic. While this form of patient partner research involvement is growing, few studies describe the process of collaboration. To address this gap, we present a case study of how university researchers worked with a patient partner on a project about mental health services for childbearing people in rural communities. The team worked together at every step, from initial study design, to reaching out to participants, reviewing the data, and writing the paper. We agreed our approach would be guided by principles such as respect for all viewpoints, speaking truth, attention to the process, and ensuring that everyone’s contributions were given equal weight. The academic researchers and the community partner identified many common themes in the data. The community partner also emphasized patient experiences of unequal treatment by health care providers. The academic researchers focused on the lack of access to perinatal mental health supports. Exploring differences in perspectives like this allowed for richer interpretation of the findings. This case study offers useful insight into the value of co-production and the important role of lived experience in improving health systems.

## Introduction

In recent years, patient partner engagement in health service research has emerged as a priority, as patient advocates and researchers recognize the value of creating epistemic bridges between knowledge producers (academics) and knowledge users (providers and patients). Several frameworks have been suggested to help guide researchers when engaging patient partners and assessing the impact of different levels of engagement on research outputs [1–4]. In British Columbia, the BC Support for People & Patient-Oriented Research & Trials (SUPPORT) Unit utilizes the Patient Engagement Framework, developed and endorsed by the Canadian Institutes of Health Research [5].

Within the field of patient partner involvement, there exists a continuum of involvement, ranging from limited/token patient inclusion in discrete parts of the research process to increasingly involved forms of engagement. On the latter side of this spectrum, an emerging form of research explores what it means to engage with patient partners beyond a normative, consultative approach, through the actual ‘co-production’ of knowledge between patients and academics. Knowledge co-production is a “value-driven approach” [2, p. 2] that recognizes the ethical and epistemological dimensions of carrying-out research as a team. Beyond exclusively measuring research as successful based on outcomes, co-analytical research also affirms the intrinsic value of involving patient collaborators in research through fostering empathy and humanizing experiences, *as well as* providing a path to concrete policy change and tangible outcomes [6].

While the field of patient engagement and co-production is growing, it remains difficult to examine in the abstract. In this paper, we focus on an application of one such integrated research team relationship, wherein a community partner with relevant lived experience partnered with a team of academic researchers to explore the topic of perinatal peer-to-peer support in rural communities.

This topic acts as a case study from which to explore how we negotiated the insider/outsider perspectives of our team. It also serves as a paragon of the productive value of incorporating lived experience into research and knowledge production, within the context of perinatal mental health services. Given our findings, we suggest five value propositions to guide the practice and method of knowledge co-production, including:

- Mutual respect for all viewpoints. This involves a commitment to recognizing and appreciating the value every collaborator brings to the endeavor. It requires appreciating unique contributions but also finding common ground.
- Rejection of assumed hierarchy. This requires privileging a hierarchy of knowledge based on lived experience with the phenomenon as opposed to a normative hierarchy based on academic qualifications.
- Attention to process. This refers to equal attention given to the *content* of our work and *how we worked together*, with neither being valued over the other.
- Commitment to truth speaking. This requires pushing the boundaries of common discourse to create room for the articulation of not only divergent theoretical perspectives but also the affective dimensions of discourse (including, for example, allowing space for the expression of emotions relating to topics at hand as well as the group process under way).
- Equivalence of contribution. The commitment to the rejection of assumed hierarchy and mutual respect for all viewpoints necessarily leads to a recognition of the equivalence of contribution. Everybody’s voice mattered equally.

## Background

In many ways, co-production is a form of knowledge production that contrasts traditional health care research, which often relies on and reproduces structures of epistemic “norms and expectations” [7]. Knowledge derived from medical theory and traditional academic work has been, and still is, privileged over knowledge rooted in patient experience, limiting epistemic authority to those in traditionally elite roles, such as academic researchers or health practitioners [4, p. 336]. Patient testimonies are often further obscured by unjust epistemic practices, including the privileging of certain strategies of expression, while other expressive styles are not recognized as rational or contextually appropriate [7]. In response, re-privileging patient narratives and allowing space for those perspectives in health care research and clinical practice is one method of countering the existing epistemic injustices embedded in many forms of traditional health research and health care [7]. Indeed, in recent years, the role of patient involvement in health research has dramatically evolved [8]. In particular there has been a turn towards collaborating with patients at all phases of the research process, including research design, participant recruitment, and knowledge dissemination [9]. This trend is reflected in the growing body of literature on co-production in mental health research in general [10–15], and specifically in perinatal mental health [16–18].

Collaborative, co-productive projects with patient partners highlight the *relationality* of research and centre intersubjectivity as the foundation of knowing in research rather than prioritizing distance between “researchers” and “participants” or maintaining traditional hierarchies of expertise [19]. Beyond patient “voice” integration, a patient co-researcher helps shape the narrative, what is kept in the paper and what is removed, and how the content is framed. This is essential to bridging the epistemological divide.

Co-production purposefully integrates patients’ experiences into health care research through the creation of an “exploratory space and a generative process” that results in multiple, intertwined forms of knowledge, values and social relations [2, p. 1]. Researchers engage the data set as diverse individuals, and then co-create meaning through engagement and dialogue with the rest of the team. This leads to socially constructed meaning that transcends what we may create as researchers working individually [20]. The versatile backgrounds and multiple perspectives offered by members engaged in the collaborative research process provide rich contexts for vibrant and rigorous analyses, disrupting the idea that traditional methodological strategies such as “saturation, triangulation, or interrater reliability” are the only meticulous approaches to dynamic, qualitative research [20].

Informed by the importance of incorporating patient experience and knowledge into research, we underwent a process of weaving together two perspectives, both of equal importance and weight, in an effort to generate new social knowledge surrounding the existence of perinatal mental health resources in rural communities. In doing so, we explicate the research practice of connecting academia with lived experience, and offer insight into how this practice of co-production contributed to a richer understanding of perinatal mental health needs in rural British Columbia.

## Methods

The project began with a virtual community forum with participants in a rural community, co-facilitated by the academic and community researchers, in order to understand some of the community-specific challenges related to mental health during the childbearing years. Rurality is an expansive description for communities outside of urban and urban-adjacent areas. Our study focused on a community of under 5000 people in a remote area of the province, approximately three hours away from the closest metropolitan centre by surface travel time. Additionally, rurality can be understood by considering the level health services locally available. The community in this study had access to continuous local hospital services with low acuity procedural care (including Cesarian section, hernia repair and appendectomy) and visiting specialist care. There is a very small Indigenous population within and adjacent to the community. Participants were passively recruited through promotional materials which were displayed across town (e.g. pharmacies, community centers) and by invitation through the patient co-lead, who was a resident of the community. In total, six participants attended the forum. Participants were asked to share their experiences of mental health challenges and access to mental health resources related to reproductive health, including: experiences of miscarriages, infertility challenges, pregnancy, birth and postpartum.

Following the community forum, a second round of participants were initially identified through purposive sampling, with the patient co-lead acting as a community facilitator. These participants were recruited based on their role in the community as social service program administrators. Snowball sampling was used to further identify potential interview participants and as a means to ensure thematic saturation. At the end of an interview, participants were asked if there was anyone else that the research team should speak to, to learn more about mental health resources in the community. Participants were interviewed virtually using an open-ended, semi-structured interview guide which probed around their organization’s values and the role of the organization in providing support to families in the childbearing years, including referring to or from medical providers. In total, 12 participants were interviewed for this stage of the project.

Each interview was attended by the co-leads and a research assistant note-taker. After each interview, research team members met to discuss the salient points from the interview. There was overarching agreement amongst team members; however, different perspectives were encountered in a few key areas. Frequently, team members would agree on the parts of the interview which were most illuminating or interesting. The interviews were transcribed by an external transcription company and were subsequently coded and analyzed separately by the patient co-lead with lived experience of mental illness (AH) and two academically trained researchers (JK and ES). The project data was analyzed separately in recognition of the different viewpoints that each of the team members brought to the project and the objective was to enable a dual perspective of the topic, from both an emic and etic point of view.

Our prioritization of hearing and documenting divergent experiences and understanding of the antenatal mental health needs of rural communities was represented in our methodological approach to the topic. That is, the research team was purposively constructed to pair a community partner (CP) with lived experience of mental illness with an academic researcher (AR) with experience with rural health research and methodology and pose questions that reflected both viewpoints and priorities, with equal value placed on each. That is, we are both researchers, but with different—complimentary—lens through which we see the phenomenon. The values proposition of *equivalence of contribution* also guided how we decided to present our findings. Instead of looking only for commonalities in interpretation of the data, we have decided to highlight differences between the ‘insider’ and ‘outside’ perspective in order to demonstrate the necessity of including both to fully understand the phenomenon. Approaching it this way has allowed us a more nuanced understanding of the value of a plurality of perspectives, including those with lived experiences of a phenomenon. It also created the need to move between an analysis of the data and a meta-analysis of the process of analysis.

We have enlisted a form of braided narrative methodology to co-create the findings and discussion. This method is often utilized in narrative, fictional work, wherein two distinct narratives are plaited together to form new meaning and develop a fuller picture of the world [21]. This approach sets up a framework for involving patient partners as research collaborators, and maintains the integrity of patient voice, as one strand of the braid, while incorporating the perspective of a traditional academic researcher as the other. It underscores the intersubjectivity of research, while requiring readers and researchers alike to hold unique, and occasionally diverging perspectives in their minds at the same time, in a productive manner [21, 22].

Our paper fits within an emerging body of literature which illuminates the process and methodology of doing research with patient partners, including how teams navigate residual/existing hierarchies and shifting balances between members of the research team, and how knowledge is co-constructed between individuals with varied backgrounds and lived experiences [23]. To this end, the analysis and write up was informed by the Guidance for Reporting Involvement of Patients and Public checklist [24]. Transparency is of vital importance to this process, as the backgrounds and experiences of each member of the team impacts their work and contributes to the rich tapestry of the co-analysis. Thus, the positionality of each research member is addressed within the methodology/discussion, and acknowledged as providing valuable locations of insight, even as certain positionalities often exist in tension with traditional health care research norms.

The CP described their role as a co-lead researcher as,

…just that, a co- lead. I was involved from the conception right through till now and ongoing. I was never “brought in” after the fact as token CP. Now this is important to note, as it validated me as a person living with a mental illness, in a world that continually tries to de- value my existence as a whole; it gave me the courage I needed to feel equal. I was being treated equal, but it was such a foreign experience for me I really had to work through some insecurities- I matter, my existence matters, I deserve to be in the space.

This paper represents the narrative of two very different demographics. As such, the experiences, and the ways we use language are very different. As the CP notes, “Both are equally deserving of respect.” Allowing the disparate voices to be represented was strategic and intentional: as the CP observed,

As a team, we had some discussion about adjusting some of my language to be more pleasing of the academic world and to ensure that we would not be denied by a journal by not sticking with status quo. To that, I said, hell no! The authenticity of my perspective and lived experience including the dialog I use to describe my findings from the data analysis must remain intact.

The CP went on to note:

… there is nothing wrong with my mentally ill plain ole vocabulary. It does not change the value of my observational perspective. It does not limit my ability to constructively, ethically and successfully analyse the data of this research.

The AR described their role slightly differently, and noted:

I saw my role as bridging the academic interpretation of the steps of the project (from conceptualization to data gathering and analysis) with the lived experience of someone with a mental illness. I recognized the importance of constant self-reflexivity and a commitment to ‘owning’ the emergence of hierarchical assumptions, usually reflected in language, if they arose. This was pretty easy to do as the CP did not hesitate to point them out (smile).

This context gave rise to the CP’s focus on hierarchy and power dynamics between providers and patients, the limitations of local services, and the importance of autonomous, patient-directed peer support. Reciprocally, the AR identified patient and provider needs within the current structure of providing care and drew out systems-level challenges including the implications of low-resource rural settings. This will be described in more detail in the findings, below.

## Findings

From an epistemological perspective, the CP interpreted the data explicitly through their lived experiences (thus findings are presented in first person voice), whereas the AR did not frame the findings in lived experience, as they did not identify with having had such experiences (thus findings are presented using third person voice). We agreed to present the findings in third person to align with academic tradition but created space for the first person narrative in the discussion section.

In this study, differences between the CP and the AR in emphasis of *importance* of findings were more prominent than differences in *interpretation*. We hypothesize that this arose from disparate social positioning within entrenched power structures in health care: the CP was a patient trying to access mental health services with a history of health system trauma and the AR was more closely collegially aligned with those providing care. Social positions further exacerbated these influences.

**Fig. 1:**
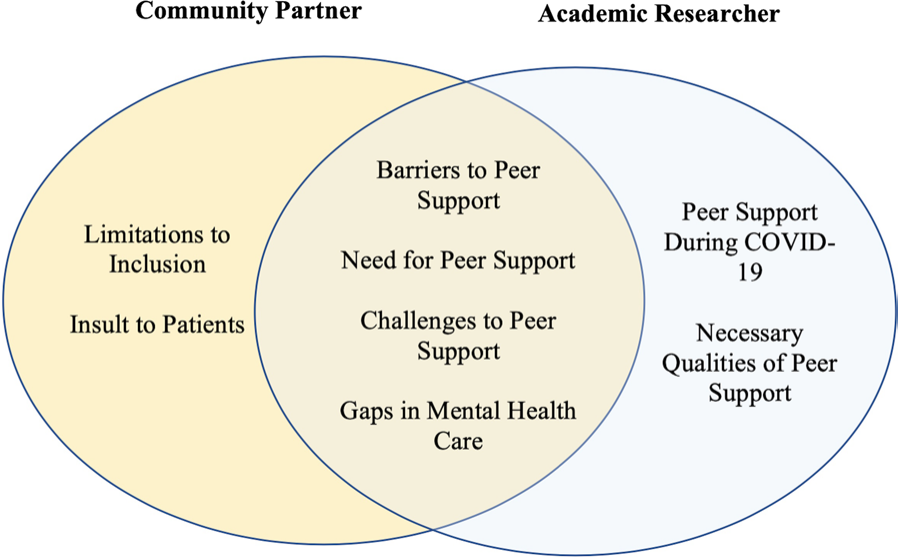
Themes by researcher orientation

Areas of overlap in interpretation included barriers to antenatal mental health care, the value of peer support, challenges to implementation, and gaps in care. Where team members most frequently disagreed or required further conversation, was related to the ways in which program administrators talked about the demographics that their organizations served and barriers to increasing engagement with more marginalized residents. Each thematic area will be described in more detail below (Fig. 1), starting with themes common to the CP and AR.

### Common themes

#### Need for peer support

There was clear agreement between both researchers that the data demonstrated a need for antenatal peer support programming to fill gaps in care for individuals with lived experience of mental health challenges. Consequently, the need for peer-support programming is a health system priority. The CP paid close attention to the potential for peer support to be a safety net for those who, despite availing themselves of more traditional counselling support, may still feel isolated. This insight was largely due to her lived experience as a patient. Both researchers recognized the value of more informal support from peers with a shared experience and the attendant reassurance that difficult times can be transient and overcome. An additional ‘value added’ quality was the ripple effect of perinatal peer support onto other areas of struggle for young families, the foundational idea being the longitudinal reference group individuals could have through the lifecycle of the family.

Although inclusion criteria for the study was being a social service program administrator or provider in the community, many participants were also parents with previous experiences of perinatal mental health challenges. The AR noted that this provided some participants with a lived experience of the need for antenatal mental health supports and grounded them from an experience-based perspective in the value of peer support. In response to their own experience of post-partum peer support, one noted,

… we hung out every single day for the entire year…she saved my life. Like, she saved me from being pretty much ready to throw in the towel. I was like ‘I'll just go back to home because I've got family there’ and it wasn't because anything was wrong with [my] relationship, it was just because I was tired and lonely, and I had no one to talk to.

Even those without direct experience, however, recognized the benefit of a peer-support model, particularly for families “who wouldn't necessarily… seek out [mental health] support.” This extended to those who may not be comfortable in a group setting due to real or perceived social differences that may lead to a sense of alienation. Even for those not experiencing social alienation, however, most participants recognized the sense of isolation that is endemic in the antenatal period (exacerbated in the time of COVID-19) and the value of relationally-based support.

#### Barriers and challenges to peer support

Both researchers noted participants’ perceptions of the lack of resources for perinatal mental health in the rural study community, precipitated by lack of funding and awareness of perinatal mental health issues. Both also recognized the need for a treatment modality guided by patients. The community partner identified lack of trust between patients and providers, and paid close attention to language as a reflection of values. For example, ‘low barrier care’ discussed by providers versus the patient priority of ‘no barrier care.’ The AR focused more on provider burnout due to the complex responsibilities of primary care providers in rural communities.

Challenges to peer support were recognized by both researchers from a structural systems perspective and a unique to rural settings perspective. The former includes the implications of health care and social service delivery norms at a bureaucratic level, including on-the-ground adherence to respect for patient privacy. This was interpreted by some to be constrictive, challenging holistic, comprehensive care. As one participant in this study noted,

There's a lot of rules there. Like we're not allowed to see these people… we're allowed to do this, we're not allowed to do that. I can share this information but… can't say [where] it came from. There’s a lot of barriers there.

Several participants also noted the lack of system acknowledgement of the importance of informal mental health supports and the difficulty of integrating these modalities into more formalized care.

Characteristics of rurality were also cited by several participants as potential barriers to peer support for some community members, namely that anonymity is often not possible simply due to social proximity and overlapping relationships. For example, a new parent may also a local teacher, shop-owner or professional in the community and interact with others through each of these roles. This is a common characteristic across rural settings and leads to difficulty maintaining confidentiality, dissuading some from participating in public groups.

#### Gaps in antenatal mental health care

Both researchers noted participants’ recognition of gaps in services due to the lack of integrated care; that is, silos in health care often limit service and provider understandings of patients’ health contexts. Likewise, both researchers noted participants’ criticisms of in-take surveys for a population facing mental health challenges, while the CP emphasized some participants observations that protocols were geared towards making things easier for staff as opposed to patients. The AR identified participants’ experiences of infrastructural challenges (such as constraints on sharing patient information between agencies, as noted above) and other service gaps (limited availability of after-hours and acute care).

From a more pragmatic perspective, a further gap in antenatal mental health care perceived by participants was the availability of after-hours acute care or routine support. There were a declining number of services providing such care (outside of emergency services through the local hospital), which many felt lead to unmet needs or, as one respondent said, “… is a major issue and really scary and concerning.” Even access to regional or provincial phone support services were noted to be limited or non-existent and, most importantly, difficult to access when they were identified. Another noted the intake barriers that exist including a provincial service that required an on-line quiz prior to access. For some, the on-line services themselves were a mediocre replacement for in-person, relationship-based care. One participant, reflecting on the contrast, noted:

And I sat with mom for four hours just talking about attachment, talking about how we love our babes, just talking about all the things that the baby was doing…. And it was interesting, in that moment, I thought why … isn't this happening every time?

Overall, forum participants expressed a desire for the normalization of mental health challenges throughout all stages of reproductive care and better integration of mental health checks into routine perinatal care. While participants were knowledgeable about mental health supports available to them in the community, long waitlists and stringent intake criteria were identified as barriers to accessing care.

Both researchers acknowledged the biggest gap in antenatal mental health care perceived by participants was the lack of diversity of modality to meet the needs of populations that differ by socioeconomic or mental health status. Several participants noted that although community programing had a mandate to be inclusive, this was often hard to achieve in part due to lack of resources to offer care to diverse groups in a way that was safe to the participants (e.g., as separate programing).

### Community partner themes

The following themes were uniquely identified by the CP and are expanded on here. The CP spoke from the perspective of someone with the lived experience of the phenomenon being discussed and did so with the strength of their voice. For this reason, we decided to leave the narrative in first person, as originally written.

#### Limits to inclusion

The thing about inclusion is it limits folks in my demographic to be included. Being mentally ill in a group setting with a majority of folks not being able to identify with and carrying the societal ingrained stigma towards mental illness, is painful. It at times is traumatizing. It can be scary and debilitating. Mentally ill folks are screaming for exclusive services and spaces, and this was noted by participants in this study:

…Probably my biggest issue… has been what I call the Circuit Mom Scenario… they are the ones at every program. They’re the mom’s who probably had a lot of support at home, probably have extended family support, probably have peers that they’re already relying on… We see them consistently at our programs. They’re our numbers. It’s not that they shouldn’t be there… it’s that we are missing those moms who aren’t comfortable walking into that scenario… who maybe aren’t comfortable with a bunch of chariots out front, and a bunch of moms in lululemon, and they feel like they’re not a part of that group…. Even though the programs obviously – they’re open to everyone.

We don’t want to sit around with “chariot row.” If you can afford a brand new chariot, matching outfits, your nails and hair professionally styled, go to the grocery store rather than the food bank, afford the ski pass, then we have very little in common.

#### Insult to patients

Findings from the research interviews demonstrated that we have a broken mental health care system. But this implies that the system was once a functioning solid complete structure. However, this has never been the case. We have systems that are hierarchal, explicitly designed leaving out the patient voices. As one participant said:

[Right now] it's just so top down, and it's so hierarchal and archaic that it actually doesn't support the needs and inclusivity of our families integrating within our community.

As we discussed earlier, this exclusion it is not only a barrier, it is an insult to patients. As if we don’t know the dire desperation of our own situations. Some made up scale is not going help the provider better understand my mental health better than I am going to.

… it was absolutely insane, because a person who's in that moment isn't filling out a quiz, you know. And it's like, who's putting these resources together? Because they don't have a hot clue [about client needs], and it's frustrating.

Participants told us about the passing of the puck between services. Unfortunately, the puck is the patient. Where the patient lands doesn’t really matter because they will still be on a wait list, for services not designed with their input, still not involved in decisions about their health and thus continuing to fall through the cracks. Now throw in a newborn baby and exhausted parent, with no history of mental illness totally unaware of how postpartum depression presents itself-sounds like a good time. Right?!

### Academic researcher themes

The following themes were uniquely identified by the AR and are expanded on here.

#### Necessary qualities

Identification of the theme ‘necessary qualities’ was noted by the AR through the lens of systems level considerations. Qualities of antenatal peer support suggested by participants ranged from macro considerations (how the program would be implemented and connectivity to existing resources), to the (micro) qualities of the program itself including the development of authentic relationships and, importantly, accessibility and flexibility. These qualities were underscored by the need for emotional safety, defined in this context as an openness to share in others’ experiences without judgement and expressed through empathy.

At a systems level, when discussing the implementation of community programs, several participants noted the importance of strategic consideration concerning where the program would ‘sit’, including how it would be developed and funded, with emphasis on the importance of diverse, grass-roots leadership. One participant noted that community members are “becoming experts in their own care.” Others emphasized the essential nature of “ground-up” development as opposed to standard “top down.”

Prioritizing client-led care was closely related, for some, to the importance of an *informal* approach to peer support that had capacity for agility in response to need. One participant described this with an example:

[A few months ago], for example, [parents] kind of took it upon themselves. They made a… connection with a bunch of moms that had babies that are around the same age. They started a walking group. And they would all get together and walk and... Yeah, totally informal, totally did it on their own.

However implemented, participants saw a key quality to peer-support being the longevity and continuity of a group starting “before babies are born” and extending to the preschool years and beyond. As one participant noted,

I think that's important to have the - the kiddos around the same age. Because I think that they feel that connection right away. And then there's some trust there. They're just kind of all on the same page then, right?

A key functional characteristic of antenatal peer support noted by several participants was *accessibility*, given the unpredictability of pregnancy or newborn parenting challenges and emotions. One recalled needing support for the middle-of-the-night struggles and the importance of “being able to call somebody not just during office hours.” They felt this need was best met by a collective as opposed to an individual to avoid burn-out. Accessibility also meant reduced barriers to access, facilitated, for example, through an open portal based on self-referral. Related to accessibility was *flexibility* in the way the program would be delivered to meet the needs and ensure the comfort of those involved in terms of meeting location, group size, and topics discussed. This included the modality of connection (in-person or virtual) with adjunctive supports such as Facebook pages. Infrastructure funding was seen by some to be essential in order to create stability for organizing and hosting meetings.

Whatever the program attributes, participants noted the importance of inter-connectivity of a peer support group with existing, more formalized mental health resources, captured by the observation that “…ideally, peer support is part of the whole spectrum of supports that are available to people.” Others emphasized the need for connection to public health and primary care, and all recognized peer support as an adjunctive modality as opposed to a replacement for clinical care.

#### Peer support during COVID-19

As noted above, most participants in this study observed that the COVID-19 pandemic accentuated the urgency for antenatal mental health supports due to family’s heighted anxiety regarding health concerns for the birther and newborn *(“I think we're all aware of the mental health and the implications that COVID could be placing on families particularly”*), uncertainty regarding protocols for the actual labour and delivery, and lack of clarity regarding safety protocols in the post-partum period. As one participant noted:

[There was] really heightened anxiety, like people were way up here. Everyone's blood pressure was way up. Like it was not just the vocalization, it was like the physiologic response of stress.

Public health protocols, during the early part of the pandemic, led to birthers’ isolation in pregnancy and families’ isolation in the post-partum period where many would usually benefit from social support. This was addressed in several ways by community mental health care providers, most notably through virtual platforms such as Facebook and Zoom.

We actually tried one thing early on in the pandemic… [a] facilitated peer support group through Facebook with two of our counselors and the local midwife.

Reflecting trends during COVID-19 across the developed world, several participants referred to the shift of program delivery from being in-person to virtual, particularly to address health-related anxiety. Although “zoom fatigue” was noted by several, one participant clearly identified the “silver lining”:

I know that everyone is talking about Zoom fatigue. I'm feeling it myself. I know my families are feeling it. [B]ut I think in some ways, there's also an advantage. [F]or example, we've been doing a book club… we meet once a week and talk about [a parenting book]. And I don't know that we would've had the turnout because we could have it at 7:30, they could put their little ones to bed and then they can join. And their partners sometimes can join.

As in many rural and remote locations, however, some participants noted the lack of connectivity due to low bandwidth and the limited effect that has on access to virtual services.

Although the shift to virtual connectivity during COVID was seen as positive by most participants, several also reflected on the inadequacy of the medium when compared to home visits both for clients but also for themselves as providers. As one noted:

Ideally, I go back to buzzing around doing home visits every day, you know. Has it changed what the role is? Yes. Has it made me reconsider if this is the role I want to be in? Absolutely. You know, home visitation was something that it means so much to me.

Several study participants highlighted the lack of attention to funding for mental health resources during COVID, pointing instead to the prioritization of funding for Personal Protective Equipment or facility adjustments to accommodate social distancing. This was seen as an oversight.

For some, COVID-19 provided a context to facilitate increased antenatal peer support due to the lack of physical space required for meetings and the additional time created by being under quarantine (or being socially cautious).

## Discussion and conclusion

The dual analysis of this study data, incorporating both emic and etic perspectives, created a richness of understanding that would not be possible through one interpretive lens. The CP’s lived experience of accessing mental health services in the perinatal period allowed insight that only one with lived experience would have. They noted evidence of limits to social inclusion through the textual analysis, driven primarily by an awareness of the social stigma surrounding mental illness and previous negative experiences predisposing patients to feeling a lack of trust. This leads to particular demographics ‘falling through the cracks’ and the tendency for services to be tailored (inadvertently or not) to those with existing resources, thus further exacerbating inequities. In response, program designs should be co-developed with marginalized populations to reflect their needs.

But the other learning from this process of co-design was the value of co-design in more comprehensively understanding the micro and macro experience of rural perinatal mental health, while maintaining a productive tension between the two. This was not easy as we bumped up against very different (usually unconscious) world views based on our divergent experiences, most evident through the use of language, both within the content of the project and more generally between academic and lay language presentation (the latter being prioritized, where possible). All members of the project team agreed on the success of the collaboration: in retrospect, we recognized it was based on five value propositions, starting with ‘mutual respect for all viewpoints.’ For the ARs, this was easily accomplished due to the clarity and authenticity of the CP’s experience. The CP experienced this as “the most kismet experience of her life.” The second value proposition was the rejection of all *assumptions* of hierarchy but acknowledging a productive hierarchy based on experience. That is, in some instances the CP was the expert (such as in the lived experience of seeking care for mental illness) while in others, the academic team were the experts (in some methodological issues and viewing the data through a health systems lens). The CP experienced the lack of hierarchy as equality with value added. This was followed by a commitment we all upheld to speaking truth, even when uncomfortable, leading to feelings of vulnerability. Truth-speaking was closely woven with authenticity, both essential to actualize the idea of ‘the whole being greater than the sum of its parts’. The AR experienced the discomfort of truth-speaking mostly through receiving honest comments from the CP that challenged their worldview, but within the context of relationship-based trust, became comfortable, familiar with and dependent on such honesty. The CP experienced truth-speaking as validation that she could exist just the way she is. She experienced commonality between the discomforts and journey to trust and comfort. A subsequent values proposition was the commitment by all team members to the attention to process. This meant that many of our weekly meetings were largely consumed with check-ins about the context of our lives and concerns and this often meant we went ‘over time’ in our meetings. For the AR, this meant we prioritized the unpredictability of our lives and circumstances over staying on a strict agenda. For the CP, this meant she could show up authentically without reprimand, judgement, or shame. Finally, ‘equivalence of contribution’ was actualized by all team members through privileging merit-based arguments, regardless of who made them.

The value of authentic co-creation of research processes and outputs cannot be over-stated: this investigation emphasized the need to break away from the tradition of assuming knowledge or expertise based on observation alone—regardless of how skillful it may be—and appreciate the wisdom of lived experience. This wisdom is best applied within the context of other forms of understanding, including the collective wisdom of communities and scientific inquiry. The inclusion of all key stakeholders is essential to actualize true understanding.

## Data Availability

The datasets generated and analyzed during the current study are not publicly available due to the small number of participants in a localized area, to prevent identification, but are available from the corresponding author on reasonable request.

## Abbreviations

CP: Community partner
AR: Academic researcher
BC: British Columbia
